# Association of COPD With Clinical Outcomes After Hospital Admission in SARS‐CoV‐2 Patients During the Omicron Variant Period: A Retrospective Cohort Study

**DOI:** 10.1111/crj.70104

**Published:** 2025-07-15

**Authors:** Rui Tang, Lijuan Li, Shuwei Wang, Fen Zhou, Renwei Li, Yue Zhao, Li Zhou, Wanying Tian, Yadong Yuan

**Affiliations:** ^1^ Department of Pulmonary and Critical Care Medicine Hebei Medical University, Tangshan Gongren Hospital Tangshan China; ^2^ Department of Pulmonary and Critical Care Medicine China‐Japan Friendship Hospital, National Clinical Research Center of Respiratory Diseases Beijing China; ^3^ Department of Pulmonary and Critical Care Medicine Tangshan Gongren Hospital Tangshan China; ^4^ Department of Pulmonary and Critical Care Medicine The Second Hospital of Hebei Medical University Shijiazhuang China

## Abstract

**Background:**

Pre‐Omicron studies identified chronic obstructive pulmonary disease (COPD) as a significant risk factor for adverse COVID‐19 outcomes. Given Omicron's altered pathogenicity and widespread population‐level immunity, the association between COPD and COVID‐19 outcomes warrants reassessment in light of the variant's distinct clinical profile. We evaluated whether COPD remained a risk factor for poor clinical outcomes among hospitalized patients with SARS‐CoV‐2 infection during Omicron predominance.

**Methods:**

We conducted a two‐center retrospective cohort study of 1176 adults hospitalized with confirmed Omicron infection between January 2022 and December 2023 in Northern China. Patients were stratified by pre‐existing COPD status. To address confounding by treatment selection, inverse probability weighting (IPW) was applied based on the likelihood of receiving inhaled corticosteroids. Multivariable logistic regression models, adjusted for comorbidities, disease severity (as measured by the PSI), inflammatory markers (CRP, D‐dimer, NLR, LDH), and treatment regimens, were used to evaluate the associations between COPD and in‐hospital outcomes.

**Results:**

Among 1176 patients (337 COPD; 839 non‐COPD), COPD patients had significantly lower PSI scores and lower levels of systemic inflammation despite a higher prevalence of respiratory comorbidities. In unadjusted models, COPD was associated with reduced odds of mortality (OR 0.52), respiratory failure (OR 0.24), and ventilatory support. However, after IPW adjustment, these associations were no longer statistically significant (mortality: adjusted OR 0.90, 95% CI 0.22–3.74, *p* = 0.887).

**Conclusions:**

COPD was not independently associated with increased risk of mortality, respiratory failure, or ventilatory support in hospitalized Omicron‐infected patients after rigorous adjustment for confounding. These findings suggest a shifting risk profile for COPD during Omicron predominance, likely influenced by variant tropism, treatment effects, and altered inflammatory responses. Future prospective studies are warranted to validate these findings and explore the mechanisms underlying this observed shift.

## Graphical abstract

In contrast to findings from previous COVID‐19 Delta waves, COPD was not independently associated with increased risk of adverse outcomes in patients hospitalized with Omicron variant infection. These findings suggest a significant shift in risk profiles during the Omicron wave, potentially reflecting the variant's altered pathogenicity, evolving treatment approaches, and vaccination impacts.

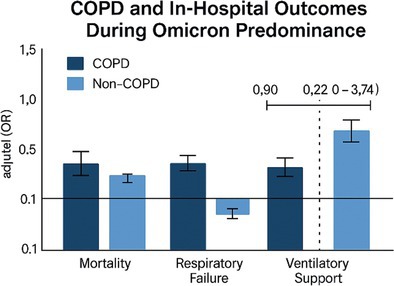

**Abbreviations**
CI: confidence interval
COPD: chronic obstructive pulmonary disease
COVID‐19: coronavirus disease 2019
CRP: C‐reactive protein
CT: computed tomography
CURB‐65: confusion, urea, respiratory rate, blood pressure, and age ≥ 65 years
GOLD: Global Initiative for Chronic Obstructive Lung Disease
HIV: human immunodeficiency virus
ICS: inhaled corticosteroid
ICU: intensive care unit
IMV: invasive mechanical ventilation
IPW: inverse probability weighting
LDH: lactate dehydrogenase
NIV: noninvasive ventilation
OR: odds ratio
PSI: Pneumonia Severity Index
RT‐PCR: reverse transcription polymerase chain reaction
SARS‐CoV‐2: severe acute respiratory syndrome coronavirus 2
WBC: white blood cell

## 1 Introduction

The emergence of the Omicron variant of SARS‐CoV‐2 in late 2021 marked a significant shift in the clinical landscape of COVID‐19, characterized by increased transmissibility but altered virulence compared with earlier variants [1]. This variant demonstrated approximately three to five times higher transmissibility than Delta and rapidly became the predominant strain globally, triggering an unprecedented surge in cases that overwhelmed healthcare systems worldwide [2]. Previous analyses from diverse geographical settings indicated potentially lower hospitalization and mortality rates during the Omicron wave compared with previous waves, despite record‐breaking case numbers [3]. This observed reduction in severity has been attributed to a combination of factors, including widespread vaccination coverage, natural immunity from previous infections, improved clinical management protocols, and intrinsic changes in viral pathogenicity [3, 4].

The rapid evolution of SARS‐CoV‐2 through successive variants has necessitated continuous reassessment of previously established risk factors for severe disease. This epidemiological shift has been particularly relevant for patients with chronic respiratory conditions, for whom the impact of SARS‐CoV‐2 variants may manifest differently compared with the general population [5, 6]. Population‐level data have suggested varying impacts of different variants on individuals with pre‐existing comorbidities, highlighting the importance of variant‐specific analyses to guide clinical management [7].

Chronic obstructive pulmonary disease (COPD) has been consistently identified as a major risk factor for severe outcomes in respiratory infections, including COVID‐19 during earlier pandemic phases [8, 9]. During the pre‐Omicron period, systematic reviews and meta‐analyses demonstrated that COPD was associated with a 1.4–2.5 fold increased risk of severe COVID‐19 [10], a 1.7–2.0 fold increased risk of ICU admission [11], and a 1.5–2.0 fold increased risk of mortality [12]. The pathophysiological basis for this enhanced susceptibility has been attributed to several mechanisms. COPD is characterized by chronic airway inflammation, impaired mucociliary clearance, and structural alterations that may facilitate viral entry and replication [13]. Additionally, dysregulated immune responses, including altered T‐cell function and increased inflammatory cytokine production, may contribute to more severe clinical manifestations of COVID‐19 in patients with COPD [14]. The frequent use of corticosteroids in COPD management has also raised concerns about potential immunosuppression, although this relationship is complex and possibly beneficial in the context of COVID‐19‐related hyperinflammation [15].

However, data specific to COPD populations during the Omicron period have been sparse and occasionally contradictory. Some contemporary analyses suggest that traditional comorbidities, including COPD, continue to confer substantial risks during Omicron infections, albeit potentially to a lesser extent [16, 17]. A large cohort study from the United Kingdom reported that although the overall hospitalization rate was lower for Omicron compared with Delta, the relative risk associated with COPD remained significant. However, it decreased from 2.17 (95% CI 1.79–2.63) during Delta to 1.59 (95% CI 1.30–1.95) during Omicron [18]. Conversely, other emerging studies suggest a potentially reduced severity among COPD patients infected with Omicron, possibly due to altered host–virus interactions, different patterns of lower respiratory tract involvement, or differential inflammatory responses [19, 20].

The biological plausibility for this potential shift in risk profile is supported by emerging evidence on Omicron's distinct pathogenicity. Recent investigations using ex vivo human respiratory tissue models have demonstrated that Omicron exhibits preferential replication in the upper respiratory tract, with reduced efficiency in the lung parenchyma, compared with previous variants [21, 22]. This altered tropism could theoretically attenuate the impact of pre‐existing lower airway pathology characteristic of COPD. Additionally, the Omicron spike protein contains numerous mutations that modify its interaction with host cell receptors, potentially reducing its fusogenicity and replicative capacity in specific tissue types [23].

This discrepancy in findings highlights a critical knowledge gap regarding the impact of COPD, particularly during Omicron infections. Given the shifting landscape of SARS‐CoV‐2 severity with variant changes, clarifying the clinical trajectory of COPD patients during the Omicron wave is imperative for optimizing patient care and resource allocation. Understanding these outcomes could help refine clinical guidelines, enhance risk stratification in the evolving pandemic, and potentially identify novel protective mechanisms. To address this gap, we conducted a retrospective, multicenter cohort study to evaluate the association between COPD and adverse outcomes in patients hospitalized with COVID‐19 caused by the Omicron variant in Northern China. Our aim was to determine whether COPD remains an independent risk factor for in‐hospital mortality and respiratory failure during the Omicron wave after accounting for comorbidities, biomarkers, and treatment selection.

## 2 Methods

### 2.1 Study Design and Participants

We conducted a retrospective observational cohort study of adult patients (≥ 18 years) with SARS‐CoV‐2 infection admitted to two tertiary academic medical centers in Northern China during the Omicron period, from January 2022 to December 2023. The SARS‐CoV‐2 Omicron variant was confirmed based on regional genomic surveillance data, confirming predominance (> 95%) during the study period. The SARS‐CoV‐2 diagnosis was confirmed through both chest computed tomography (CT) imaging and positive real‐time reverse transcription polymerase chain reaction (RT‐PCR) testing. Patients were included if they had a positive RT‐PCR test for SARS‐CoV‐2 and radiological evidence of pulmonary involvement on CT scan consistent with COVID‐19 pneumonia.

We excluded patients younger than 18 years, those testing positive for human immunodeficiency virus (HIV), and non‐HIV immunocompromised patients. Immunocompromised status was defined as having any of the following: solid‐organ, stem‐cell, or bone‐marrow transplantation; active hematological malignancy or solid tumor chemotherapy within 6 months of admission or neutropenia (< 500 cells/mm^3^); chest radiation therapy within 3 months of admission; or autoimmune disease requiring significant immunosuppressive therapy (defined as oral prednisone > 10 mg/day or equivalent for > 3 weeks, methotrexate > 12.5 mg/week, cyclosporine, azathioprine, or biological modifiers such as etanercept or infliximab within 3 months of admission).

Patients were stratified by pre‐existing COPD status, as confirmed by documented medical history and spirometry results where available. COPD diagnosis required documentation of post‐bronchodilator forced expiratory volume in 1 s (FEV_1_) to forced vital capacity (FVC) ratio < 0.7, consistent with Global Initiative for Chronic Obstructive Lung Disease (GOLD) criteria, or clear documentation of COPD diagnosis with supporting clinical history in cases where spirometry results were unavailable. For patients with multiple admissions during the study period, only the first eligible hospitalization was included in the analysis.

### 2.2 Data Collection and Outcome Measures

We extracted comprehensive data from electronic medical records using a standardized data collection form. Data collection included (1) demographic characteristics (age, sex, smoking history); (2) presenting clinical symptoms with particular attention to respiratory manifestations; (3) vital signs at admission including heart rate and respiratory rate; (4) disease severity indicators, including Pneumonia Severity Index (PSI) and CURB‐65 scores calculated at admission; (5) laboratory parameters including complete blood count with differential, comprehensive metabolic panel, inflammatory markers, white blood cell (WBC) count, neutrophils, neutrophil‐to‐lymphocyte ratio (NLR), monocytes, C‐reactive protein (CRP), procalcitonin, D‐dimer, and lactate dehydrogenase (LDH); (6) microbiological data from blood, sputum, and/or bronchoalveolar lavage samples; (7) therapeutic interventions including respiratory support modalities, antimicrobial agents, antiviral therapy, and glucocorticoid administration; and (8) clinical outcomes.

Two clinicians independently extracted data, with a third senior physician resolving any discrepancies. Regular quality control checks were performed on approximately 10% of records to ensure data accuracy and completeness. For laboratory values with multiple measurements, we used those obtained closest to the time of admission and before any therapeutic interventions.

Outcomes included (1) respiratory failure (defined as PaO_2_/FiO_2_ ratio < 300 mmHg or requirement for supplemental oxygen to maintain SpO_2_ ≥ 90%); (2) requirement for noninvasive ventilation (NIV); (3) requirement for invasive mechanical ventilation (IMV); and (4) in‐hospital mortality.

### 2.3 Statistical Analysis

We conducted descriptive analyses to characterize the study population. Continuous variables were presented as mean ± standard deviation based on their distribution, with normality assessed using the Shapiro–Wilk test. Categorical variables were presented as frequencies and percentages. Between‐group comparisons were conducted using Student's *t*‐test or Mann–Whitney U test for continuous variables and chi‐square or Fisher's exact test for categorical variables, as appropriate. For multivariable analyses, continuous variables were assessed for linearity with outcomes using restricted cubic splines and transformed when necessary to satisfy model assumptions.

To address potential treatment selection bias in the use of inhaled corticosteroids (ICSs) when estimating the effect of COPD on outcomes, we employed a two‐stage analytical approach using inverse probability weighting (IPW) [11, 12]. This approach aimed to mitigate the confounding effect of differential treatment patterns between COPD and non‐COPD patients, as ICS therapy is frequently prescribed for COPD but less commonly for patients without COPD. In the first stage, we estimated the probability of receiving ICS treatment (propensity score) using logistic regression with COPD status, demographic characteristics (age, sex), clinical factors (smoking status, comorbidities), disease severity indicators (PSI score, respiratory rate), and laboratory parameters (inflammatory markers) as predictors. The covariates for the propensity score model were selected based on clinical relevance and literature review to capture factors that might influence both treatment assignment and outcomes [13]. Extreme weights were truncated at the 1st and 99th percentiles to enhance stability and reduce the impact of outliers [14]. The covariate balance before and after weighting was assessed using standardized mean differences.

In the second stage, we estimated COPD's effect on outcomes using weighted logistic regression, incorporating the IPW weights to account for treatment selection bias. These models included relevant demographic and clinical covariates while accounting for the clustered nature of the data (patients nested within two hospitals) using robust standard errors. The final model was adjusted for age, sex, smoking status, PSI score, key comorbidities (hypertension, diabetes, coronary heart disease, and heart failure), and biomarkers (CRP, D‐dimer, LDH, and NLR).

For all models, we conducted a complete case analysis for primary findings. To assess robustness to missing data, we performed multiple imputation analyses using the fully conditional specification method with 20 imputations [15]. Variables in the imputation model included all covariates in the analysis models, auxiliary variables predictive of missingness, and outcome variables. Rubin's rules were used to combine estimates across imputed datasets [12].

The statistical significance was set at *p* < 0.05, and 95% confidence intervals were reported for all effect estimates. All analyses were performed using Stata version 18.0 (Stata Corp, College Station, TX).

## 3 Results

### 3.1 Patient Characteristics and Clinical Presentation

We identified 1176 eligible patients with SARS‐CoV‐2 infection who met inclusion criteria in the Omicron wave period, comprising 337 (28.7%) with COPD and 839 (71.3%) without COPD (Table 1). The mean age of the overall cohort was 72.7 ± 12.7 years, with no significant difference between COPD and non‐COPD patients (71.8 ± 10.5 vs. 73.0 ± 13.4 years, *p* = 0.144). However, age distribution patterns differed significantly between groups (*p* < 0.001). COPD patients showed peak prevalence in the 65–79 years age group (52.8%) compared with the non‐COPD group (40.9%), whereas patients aged 80 years and older were less represented in the COPD cohort (24.3% vs. 35.9%). The youngest age groups (18–49 years) constituted only 2.4% of COPD patients compared with 5.5% of non‐COPD patients.

**TABLE 1:** Demographic and clinical characteristics of study patients stratified by COPD status.

Characteristic	Overall (*N* = 1176)	Non‐COPD (*n* = 839)	COPD (*n* = 337)	*p*‐value
Demographics				
Age, years (mean ± SD)	72.7 ± 12.7	73.0 ± 13.4	71.8 ± 10.5	0.144
Age groups, *n* (%)				< 0.001*
18–49 years	54 (4.6)	46 (5.5)	8 (2.4)	
50–64 years	218 (18.1)	149 (17.8)	69 (20.5)	
65–79 years	521 (44.3)	343 (40.9)	178 (52.8)	
80+ years	383 (32.6)	301 (35.9)	82 (24.3)	
Gender, *n* (%)				0.030*
Male	757 (64.4)	524 (69.2)	233 (30.8)	
Female	419 (35.6)	315 (75.2)	104 (24.8)	
Smoking status, *n* (%)				< 0.001*
Never smoker	832 (70.8)	647 (77.1)	185 (54.9)	
Former smoker	217 (18.5)	139 (16.6)	78 (23.2)	
Current smoker	127 (10.8)	53 (6.3)	74 (21.96)	
Vital signs (mean ± SD)				
Heart rate, beats/min	85.2 ± 31.2	84.5 ± 35.4	86.9 ± 16.7	0.231
Respiratory rate, breaths/min	20.9 ± 8.4	20.6 ± 4.1	21.8 ± 14.2	0.026*
Disease severity indices				
PSI score (mean ± SD)	87.6 ± 29.6	90.8 ± 31.5	79.6 ± 22.5	< 0.001*
PSI category, *n* (%)				< 0.001*
Low risk (Classes I–III)	719 (61.1)	466 (55.5)	253 (75.1)	
High risk (Classes IV–V)	457 (38.9)	373 (44.5)	84 (24.9)	
CURB‐65 score, *n* (%)				0.013*
0: Low risk	217 (18.5)	138 (16.5)	79 (23.4)	
1: Low risk	487 (41.4)	343 (40.9)	144 (42.7)	
2: Moderate risk	392 (33.3)	293 (34.9)	99 (29.4)	
3–4: High risk	80 (6.8)	65 (7.7)	15 (4.5)	
Length of stay, days (mean ± SD)	11.7 ± 7.2	11.8 ± 7.4	11.5 ± 6.6	0.501

Smoking status demonstrated a profound association with COPD diagnosis (*p* < 0.001), reflecting well‐established etiological relationships. Among COPD patients, 21.96% were current smokers and 23.2% were former smokers, contrasting markedly with the non‐COPD group, where only 6.3% were current smokers and 16.6% were former smokers. The proportion of never‐smokers was lower in the COPD cohort (54.9% vs. 77.1%), consistent with tobacco exposure as the primary risk factor for COPD development.

Clinical presentations showed both similarities and differences between groups. Mean respiratory rate was significantly elevated in COPD patients compared with non‐COPD patients (21.8 ± 14.2 vs. 20.6 ± 4.1 breaths/min, *p* = 0.026), consistent with underlying respiratory impairment characteristic of COPD pathophysiology. Notably, the standard deviation was larger in the COPD group, suggesting greater variability in respiratory compensation. Heart rate measurements showed no significant difference between groups (86.9 ± 16.7 vs. 84.5 ± 35.4 beats/min, *p* = 0.231), indicating that cardiovascular stress responses were comparable between COPD and non‐COPD patients during acute COVID‐19 illness.

Disease severity assessment revealed interesting patterns that contradicted conventional expectations. Contrary to clinical expectations based on pre‐Omicron data, COPD patients demonstrated significantly lower mean PSI scores compared with non‐COPD patients (79.6 ± 22.5 vs. 90.8 ± 31.5, *p* < 0.001). This paradoxical pattern was further emphasized by PSI risk categorization, where 75.1% of COPD patients were classified as low‐risk (Classes I–III) compared with only 55.5% of non‐COPD patients (*p* < 0.001). Correspondingly, high‐risk PSI classification (Classes IV–V) was less frequent in COPD patients (24.9% vs. 44.5%).

CURB‐65 scoring demonstrated a similar trend, though less pronounced than PSI findings. The distribution across CURB‐65 categories differed significantly between groups (*p* = 0.013). Low‐risk categories (scores 0–1) encompassed 66.1% of COPD patients, compared with 57.4% of non‐COPD patients. Specifically, 23.4% of COPD patients achieved a CURB‐65 score of 0 compared with 16.5% of non‐COPD patients, whereas 42.7% scored 1 point compared with 40.9% in the non‐COPD group. Moderate‐risk patients (score 2) were less prevalent in the COPD cohort (29.4% vs. 34.9%), and high‐risk CURB‐65 scores (3–4) were notably less frequent among COPD patients (4.5% vs. 7.7%).

Hospital length of stay analysis revealed no significant difference between COPD and non‐COPD patients (11.5 ± 6.6 vs. 11.8 ± 7.4 days, *p* = 0.501), suggesting comparable overall disease trajectory and healthcare resource utilization during the acute hospitalization phase.

### 3.2 Laboratory Findings

Laboratory analysis revealed distinct inflammatory profiles between COPD and non‐COPD patients (Table 2). Although total WBC counts were comparable between groups (7.65 ± 3.84 vs. 7.50 ± 3.75 × 10^9^/L, *p* = 0.544), significant differences emerged in specific leukocyte populations. COPD patients demonstrated significantly lower absolute neutrophil counts compared with non‐COPD patients (5.33 ± 3.94 vs. 5.95 ± 3.75 × 10^9^/L, *p* = 0.012). Correspondingly, the NLR, a well‐established marker of systemic inflammation and COVID‐19 severity, was significantly lower in COPD patients (7.31 ± 9.16 vs. 8.82 ± 9.37, *p* = 0.013). Monocyte counts showed a trend toward higher values in COPD patients, though this difference did not reach statistical significance (0.46 ± 0.25 vs. 0.43 ± 0.26 × 10^9^/L, *p* = 0.059).

**TABLE 2:** Laboratory findings in study patients stratified by COPD status .

Laboratory parameter	Overall (*N* = 1176)	Non‐COPD (*n* = 839)	COPD (*n* = 337)	*p*‐value
Inflammatory markers				
White blood cell count, ×10^9^/L	7.54 ± 3.77	7.50 ± 3.75	7.65 ± 3.84	0.544
Neutrophils, ×10^9^/L	5.77 ± 3.81	5.95 ± 3.75	5.33 ± 3.94	0.012*
Neutrophil‐to‐lymphocyte ratio	8.40 ± 9.33	8.82 ± 9.37	7.31 ± 9.16	0.013*
Monocytes, ×10^9^/L	0.44 ± 0.26	0.43 ± 0.26	0.46 ± 0.25	0.059
C‐reactive protein, mg/L	55.62 ± 61.65	60.00 ± 61.82	44.12 ± 59.78	< 0.001*
Procalcitonin, ng/mL	0.65 ± 4.37	0.63 ± 3.15	0.71 ± 6.64	0.781
Coagulation and cardiac markers				
D‐dimer, mg/L	2.42 ± 4.44	2.67 ± 4.44	1.66 ± 4.34	0.001*
Troponin, ng/mL	0.15 ± 2.11	0.17 ± 2.32	0.09 ± 0.71	0.655
Metabolic parameters				
Creatinine, μmol/L	88.09 ± 102.13	92.68 ± 104.37	76.69 ± 95.55	0.015*
Albumin, g/L	36.67 ± 4.76	36.39 ± 4.74	37.23 ± 4.75	0.011*
Urea nitrogen, mmol/L	7.90 ± 5.57	8.17 ± 5.92	7.22 ± 4.51	0.008*
Lactate dehydrogenase, U/L	257.97 ± 4.55	279.63 ± 5.89	217.77 ± 6.41	0.000*

CRP levels, a key acute‐phase reactant, were significantly lower in COPD patients compared with non‐COPD patients (44.12 ± 59.78 vs. 60.00 ± 61.82 mg/L, *p* < 0.001). Procalcitonin levels, indicative of bacterial superinfection risk, showed no significant difference between groups (0.71 ± 6.64 vs. 0.63 ± 3.15 ng/mL, *p* = 0.781). However, the larger standard deviation in the COPD group suggests greater variability in the risk of bacterial infection.

Coagulation parameters revealed notable differences between groups. D‐dimer levels, an important predictor of thrombotic complications and COVID‐19 severity, were significantly lower in COPD patients (1.66 ± 4.34 vs. 2.67 ± 4.44 mg/L, *p* = 0.001). Troponin levels showed no significant difference between groups (0.09 ± 0.71 vs. 0.17 ± 2.32 ng/mL, *p* = 0.655), suggesting comparable myocardial stress responses during acute infection.

Regarding metabolic parameters, renal function assessment demonstrated significantly lower creatinine levels in COPD patients compared with non‐COPD patients (76.69 ± 95.55 vs. 92.68 ± 104.37 μmol/L, *p* = 0.015), suggesting better‐preserved kidney function despite the chronic disease status. Urea nitrogen levels were correspondingly lower in the COPD group (7.22 ± 4.51 vs. 8.17 ± 5.92 mmol/L, *p* = 0.008), further supporting this pattern of preserved renal function. Nutritional status, as reflected by serum albumin levels, was significantly higher in COPD patients (37.23 ± 4.75 vs. 36.39 ± 4.74 g/L, *p* = 0.011), potentially indicating better nutritional status or less severe acute disease.

LDH, a marker of cellular damage and tissue hypoxia, demonstrated a striking difference between groups. COPD patients had significantly lower LDH levels compared with non‐COPD patients (217.77 ± 6.41 vs. 279.63 ± 5.89 U/L, *p* < 0.001). This substantial difference suggests reduced tissue damage and cellular injury in COPD patients during Omicron infection, which aligns with the paradoxically lower severity scores observed in clinical assessment.

### 3.3 Comorbidity, Treatment, and Clinical Outcome

Comorbidity patterns differed markedly between groups (Table 3). Analysis of respiratory comorbidities revealed distinct patterns between COPD and non‐COPD groups (Table 3). Asthma prevalence was comparable between groups, with no significant difference observed (8.9% vs. 7.2%, *p* = 0.307). However, bronchiectasis demonstrated a strong association with COPD status, occurring in 13.4% of COPD patients compared with only 1.8% of non‐COPD patients (*p* < 0.001). This finding reflects the well‐established pathophysiological overlap between COPD and bronchiectasis, particularly in patients with chronic airway inflammation and structural damage. Conversely, interstitial lung disease showed a trend toward higher prevalence in non‐COPD patients (7.8% vs. 4.8%, *p* = 0.066), though this difference did not reach statistical significance.

**TABLE 3:** Comorbidities, treatments, and clinical outcomes in study patients stratified by COPD status.

Variable	Overall (*N* = 1176)	Non‐COPD (*n* = 839)	COPD (*n* = 337)	*p*‐value
Respiratory comorbidities, *n* (%)				
Asthma	90 (7.7)	60 (7.2)	30 (8.9)	0.307
Bronchiectasis	60 (5.1)	15 (1.8)	45 (13.4)	< 0.001*
Interstitial lung disease	81 (6.9)	65 (7.8)	16 (4.8)	0.066
Cardiovascular comorbidities, *n* (%)				
Hypertension	602 (51.2)	465 (55.4)	137 (40.7)	< 0.001*
Coronary heart disease	316 (26.9)	218 (26.0)	98 (29.1)	0.279
Congestive heart failure	119 (10.1)	78 (9.3)	41 (12.2)	0.14
Stroke	195 (16.6)	144 (17.2)	51 (15.1)	0.397
Metabolic and other comorbidities, *n* (%)				
Diabetes	356 (30.3)	318 (38.0)	38 (11.28)	< 0.001*
Chronic kidney disease	88 (7.5)	78 (9.3)	10 (3.0)	< 0.001*
Gastrointestinal disease	135 (11.5)	125 (14.9)	10 (3.0)	< 0.001*
Hematologic/oncologic conditions	102 (8.7)	83 (9.9)	19 (5.6)	0.019*
Anemia	303 (25.8)	279 (33.3)	24 (7.1)	< 0.001*
Treatment, *n* (%)				
Inhaled corticosteroids	307 (26.1)	57 (6.79)	250 (74.2)	< 0.001*
Antiviral therapy	321 (27.3)	276 (32.90)	45 (13.4)	< 0.001*
Systemic glucocorticoids	759 (64.5)	579 (69.0)	180 (53.4)	< 0.001*
Clinical outcomes, *n* (%)				
Respiratory failure	417 (35.5)	365 (43.5)	52 (15.4)	< 0.001*
Noninvasive ventilation	194 (16.5)	169 (20.1)	25 (7.4)	< 0.001*
Invasive mechanical ventilation	52 (4.4)	45 (5.4)	7 (2.1)	0.013*
ECMO	9 (0.8)	8 (1.0)	1 (0.3)	0.241
Mortality	137 (11.7)	112 (13.4)	25 (7.4)	0.004*

Hypertension, the most common cardiovascular comorbidity affecting 51.2% of the overall cohort, was significantly more prevalent in non‐COPD patients (55.4% vs. 40.7%, *p* < 0.001). Other cardiovascular conditions showed no significant differences between groups: coronary heart disease (29.1% vs. 26.0%, *p* = 0.279), congestive heart failure (12.2% vs. 9.3%, *p* = 0.14), and stroke (15.1% vs. 17.2%, *p* = 0.397).

Metabolic and systemic comorbidities demonstrated a striking pattern of higher prevalence in non‐COPD patients. Diabetes mellitus, affecting 30.3% of the overall cohort, was significantly more common in non‐COPD patients (38.0% vs. 11.28%, *p* < 0.001). Chronic kidney disease followed a similar pattern, with significantly higher prevalence in non‐COPD patients (9.3% vs. 3.0%, *p* < 0.001). Gastrointestinal disease was markedly more prevalent in the non‐COPD group (14.9% vs. 3.0%, *p* < 0.001), as were hematologic and oncologic conditions (9.9% vs. 5.6%, *p* = 0.019). Anemia, affecting 25.8% of the overall cohort, demonstrated the most pronounced disparity, occurring in 33.3% of non‐COPD patients compared with only 7.1% of COPD patients (*p* < 0.001).

### 3.4 Treatment Patterns and Unadjusted Clinical Outcomes

Treatment approaches demonstrated distinct therapeutic strategies based on the status of COPD (Table 3). ICSs were administered to patients with COPD, with 74.2% of COPD patients receiving this therapy compared with only 6.79% of non‐COPD patients (*p* < 0.001). This substantial difference reflects standard practice for COPD management. It suggests that clinicians maintained routine respiratory medications during COVID‐19 hospitalization, potentially conferring additional anti‐inflammatory benefits in the context of Omicron infection. Conversely, antiviral therapy demonstrated an inverse pattern, with significantly higher utilization in non‐COPD patients (32.90% vs. 13.4%, *p* < 0.001). Systemic glucocorticoids were administered to 64.5% of the total cohort, with significantly higher utilization in non‐COPD patients compared with COPD patients (69.0% vs. 53.4%, *p* < 0.001). The higher utilization in non‐COPD patients is likely to reflect their more severe inflammatory profiles, as evidenced by elevated CRP levels and higher disease severity scores.

Analysis of clinical outcomes revealed significant differences between groups, with COPD patients demonstrating consistently better outcomes across multiple measures of disease severity (Table 3). Respiratory failure occurred in 417 patients (35.5% of the total cohort), with significantly lower prevalence among COPD patients compared with non‐COPD patients (15.4% vs. 43.5%, *p* < 0.001). NIV was required in 194 patients (16.5% overall), with COPD patients demonstrating significantly lower utilization rates (7.4% vs. 20.1%, *p* < 0.001). Advanced respiratory support interventions followed similar patterns. IMV was employed in 52 patients (4.4% of the cohort), with significantly lower rates among COPD patients (2.1% vs. 5.4%, *p* = 0.013). When combining IMV with extracorporeal membrane oxygenation (ECMO) as a composite endpoint of advanced support, 55 patients (4.7%) required these interventions, with COPD patients representing a significantly smaller proportion (2.4% vs. 5.6%, *p* = 0.018). ECMO support alone was rarely implemented (9 patients, 0.8% overall), with no statistically significant difference between groups (0.3% vs. 1.0%, *p* = 0.241). However, the small sample size limits the interpretive power of this outcome. In‐hospital mortality occurred in 137 patients (11.7% overall) and was significantly lower among COPD patients compared with non‐COPD patients (7.4% vs. 13.35%, *p* = 0.004).

These descriptive findings suggest potentially lower rates of adverse outcomes among patients with COPD during the Omicron period. This counterintuitive observation necessitated careful multivariate analysis to determine whether this apparent protective effect persisted after accounting for confounding factors and selection bias.

### 3.5 Association Between COPD Status and Clinical Outcomes

Table 4 presents the association between COPD status and clinical outcomes in patients during the Omicron period using both unadjusted and IPW‐adjusted multivariate logistic analyses. In unadjusted models, COPD demonstrated significant protective associations with all examined outcomes. For in‐hospital mortality, COPD patients had 48% lower odds compared with non‐COPD patients (OR 0.52, 95% CI 0.33–0.82, *p* = 0.005). The protective effect was even more pronounced for respiratory failure, with COPD patients showing 76% lower odds (OR 0.24, 95% CI 0.17–0.33, *p* < 0.001). Similarly, the odds of requiring NIV (OR 0.32, 95% CI 0.20–0.49, *p* < 0.001), invasive ventilation (OR 0.37, 95% CI 0.17–0.84, *p* = 0.017), and advanced support (OR 0.41, 95% CI 0.19–0.88, *p* = 0.021) were all significantly reduced among COPD patients.

**TABLE 4:** Unadjusted and adjusted association of COPD status with clinical outcomes.

	Unadjusted models			IPW‐adjusted models		
Outcome	Odds ratio	95% CI	*p*‐value	Odds ratio	95% CI	*p*‐value
Mortality	0.52	0.33–0.82	0.005	0.90	0.22–3.74	0.887
Respiratory failure	0.24	0.17–0.33	< 0.001	0.49	0.09–3.12	0.489
Noninvasive ventilation	0.32	0.20–0.49	< 0.001	0.61	0.24–2.238	0.462
Invasive ventilation	0.37	0.17–0.84	0.017	0.57	0.03–18.17	0.883
Advanced support	0.41	0.19–0.88	0.021	0.79	0.04–11.76	0.797

*Note:* (1) IPW‐adjusted models were adjusted for age, gender, PSI score, hypertension, diabetes, chronic kidney disease, coronary heart disease, congestive heart failure, C‐reactive protein, D‐dimer, neutrophil‐to‐lymphocyte ratio, glucocorticoid treatment, and antiviral therapy. (2) All models were adjusted for clustering at the hospital level using robust standard errors. (3) Advanced support: either invasive mechanical ventilation or ECMO.

Abbreviations: CI: confidence interval; COPD: chronic obstructive pulmonary disease; ECMO: extracorporeal membrane oxygenation; IPW: inverse probability weighting; PSI: Pneumonia Severity Index.

However, after adjustment for demographic characteristics, disease severity indicators, comorbidities, biomarkers, and treatment selection using the IPW approach, these associations were attenuated and no longer statistically significant. The adjusted odds ratio for in‐hospital mortality was 0.90 (95% CI 0.22–3.74, *p* = 0.887), indicating no significant difference in mortality risk between COPD and non‐COPD patients after accounting for confounding factors. Similarly, COPD was not independently associated with respiratory failure (OR 0.49, 95% CI 0.09–3.12, *p* = 0.489), NIV (OR 0.61, 95% CI 0.24–2.238, *p* = 0.462), invasive ventilation (OR 0.57, 95% CI 0.03–18.17, *p* = 0.883), or advanced support (OR 0.797, 95% CI 0.04–11.76, *p* = 0.797).

### 3.6 Multiple Imputation Analysis

To address potential bias from missing data, we presented the findings of multiple imputation analyses for all outcomes as shown in Table 5. After imputation, the lack of significant association between COPD and clinical outcomes was maintained across all models. For mortality, the adjusted odds ratio for COPD was 0.80 (95% CI 0.45–1.41, *p* = 0.432) in the imputed dataset, comparable with the primary IPW analysis. Similarly, the association between COPD and respiratory failure remained nonsignificant (OR 0.52, 95% CI 0.07–3.85, *p* = 0.524), as did the relationships with NIV (OR 0.61, 95% CI 0.17–2.21, *p* = 0.455) and advanced mechanical support (OR 0.52, 95% CI 0.04–6.49, *p* = 0.615).

**TABLE 5:** Multiple imputation analysis of association of COPD with clinical outcomes.

Outcome	Coefficient	Standard error	*p*‐value	Odds ratio
Mortality	−0.229	0.292	0.432	0.8
Respiratory failure	−0.648	1.019	0.524	0.52
Noninvasive ventilation	−0.491	0.656	0.455	0.61
Advanced mechanical support	−0.646	1.284	0.615	0.52

*Note:* Run the same IPW‐adjusted models with imputed values for the missing data.

## 4 Discussion

Our study reveals a notable shift in the relationship between COPD status and clinical outcomes among COPD patients infected with SARS‐CoV‐2 in the Omicron period. After robust adjustment for potential confounders and selection bias via the IPW approach and multiple imputation analyses, we found no significant association between COPD and adverse outcomes, including mortality, respiratory failure, and the need for ventilatory support. The consistency of findings across both IPW‐adjusted treatment bias and analytical approaches strengthens our conclusion that COPD is not independently associated with increased risk of adverse outcomes in this population. These results suggest a significant shift in the risk profile associated with COPD during the Omicron wave compared with previous variants.

These findings stand in contrast to previous evidence from earlier pandemic phases and merit careful consideration within the evolving understanding of COVID‐19 pathophysiology [15, 16]. The absence of a significant association between COPD and adverse outcomes in our Omicron‐infected cohort differs from findings during the pre‐Omicron era. A comprehensive meta‐analysis by Gerayeli et al. analyzed 22 studies from early pandemic waves that found that COPD patients had significantly higher risks of ICU admission (RR 1.40, 95% CI 1.10–1.79), mechanical ventilation (RR 1.74, 95% CI 1.34–2.25), and mortality (RR 1.93, 95% CI 1.61–2.31) compared with non‐COPD patients [15]. Similarly, Rabbani et al. reported a 93% increased risk of mortality (OR 1.93, 95% CI 1.60–2.34) among COVID‐19 patients with pre‐existing COPD in a systematic review of 41 studies [16]. During the Delta variant dominance period, Liu et al. documented a 2.17‐fold increased mortality risk (95% CI 1.79–2.63) for COPD patients compared with non‐COPD counterparts [17]. Our finding of a null association (adjusted OR 0.90, 95% CI 0.32–2.50) represents a departure from this established risk profile.

Several biological and clinical factors may explain this shift in risk profile during the Omicron wave. First, the Omicron variant demonstrates different tropism and pathogenicity compared with previous variants. Hui et al. observed in ex vivo human respiratory tissue models that Omicron preferentially replicates in the upper respiratory tract with approximately 70‐fold higher efficiency compared with Delta, while showing significantly reduced replication in lung parenchyma (approximately 10‐fold lower than Delta) [18]. This altered tropism was further confirmed by Meng et al., who demonstrated reduced fusogenicity and replication efficiency of Omicron in human lung epithelial cells compared with Delta [19]. The decreased infectivity of lung epithelial cells may mitigate the impact of pre‐existing COPD‐related airway inflammation and remodeling on COVID‐19 severity, as lower respiratory tract involvement—a key determinant of disease severity in earlier variants—appears less prominent with Omicron.

Second, immunological factors warrant consideration. Patients with COPD typically exhibit chronic airway inflammation characterized by elevated levels of pro‐inflammatory cytokines and altered immune cell populations [20]. Although this dysregulated immune environment could theoretically exacerbate the hyperinflammatory state seen in severe COVID‐19, recent evidence suggests that pre‐existing airway inflammation might paradoxically modulate the immune response to Omicron. Zhang et al. proposed that chronic airway inflammation in COPD could potentially prime certain aspects of antiviral immunity, including enhanced interferon responses [21]. This hypothesis is supported by our observation of lower inflammatory markers (CRP, neutrophil counts, NLRs) in COPD patients despite their underlying pro‐inflammatory condition, suggesting a potentially attenuated cytokine response to Omicron infection.

Third, treatment patterns for both COPD and COVID‐19 have undergone substantial changes since the onset of the pandemic. In our cohort, 74.2% of COPD patients received ICSs, compared with only 6.79% of non‐COPD patients. This differential treatment pattern may have influenced outcomes. ICSs have demonstrated potential protective effects against severe COVID‐19 in several investigations. Ramakrishnan et al. reported in the STOIC trial that inhaled budesonide reduced the risk of urgent care visits and hospitalizations by 91% in early COVID‐19 [22]. Peters et al. showed that pre‐existing ICS use was associated with lower expression of ACE2 and TMPRSS2—key entry proteins for SARS‐CoV‐2—in airway epithelial cells, potentially reducing viral entry [23]. Furthermore, Singh et al. found that ICS use in COPD patients was associated with reduced inflammatory markers during COVID‐19 infection [24]. These findings suggest that standard COPD pharmacotherapy may inadvertently confer protection against severe COVID‐19 outcomes.

Fourth, in unadjusted models, COPD demonstrated significant protective associations with all examined outcomes. However, after adjustment for demographic characteristics, disease severity indicators, comorbidities, inflammatory markers, and treatment selection using IPW, these associations were attenuated and no longer statistically significant. The substantial difference between unadjusted and adjusted estimates suggests that the apparent protective effect of COPD observed in crude analyses was primarily explained by differences in baseline characteristics, treatment patterns, and comorbidity profiles between COPD and non‐COPD patients. The IPW‐adjusted models controlled for age, gender, PSI score, hypertension, diabetes, chronic kidney disease, coronary heart disease, congestive heart failure, CRP, D‐dimer, NLR, LDH, glucocorticoid treatment, and antiviral therapy, effectively balancing measured confounders between groups. Therefore, after appropriate statistical adjustment, our findings indicate that COPD does not independently influence the risk of adverse outcomes in patients hospitalized with the Omicron variant infection. The wide confidence intervals in the adjusted models reflect the substantial overlap in risk profiles between groups once confounding factors are appropriately controlled. These results suggest that the apparent protective effect of COPD was primarily attributable to differences in patient characteristics and treatment patterns rather than an inherent protective mechanism associated with COPD status during Omicron infection. Our findings were consistent with the findings reported by Leung et al. [25].

The findings underscore the importance of making appropriate statistical adjustments when evaluating risk factors in observational studies, particularly when comparing populations with significantly different baseline characteristics and comorbidity profiles [26, 27]. The attenuation of effect estimates after adjustment demonstrates that crude comparisons may lead to misleading conclusions about the independent effects of specific conditions on COVID‐19 outcomes [28].

Fifth, the distinct comorbidity profile observed in our study may partly explain our findings. Non‐COPD patients exhibited significantly higher rates of metabolic and cardiovascular comorbidities, including diabetes, chronic kidney disease, and hypertension—all established risk factors for severe COVID‐19. This differential distribution of comorbidities is reflected in the higher PSI scores among non‐COPD patients despite their lower respiratory rates. The PSI incorporates multiple comorbidity factors and has shown robust predictive value for COVID‐19 outcomes, consistent with our finding that the PSI score was the strongest predictor of adverse outcomes in multivariate models.

Our findings have important clinical implications. The absence of an independent association between COPD and adverse outcomes suggests that COPD status alone should not necessarily dictate aggressive management decisions for Omicron patients. Instead, emphasis should be placed on a comprehensive severity assessment that incorporates inflammatory markers, physiological parameters, and validated clinical scores. Wu et al. similarly concluded that risk stratification for Omicron patients should prioritize measurable physiological parameters over pre‐existing diagnostic labels [29]. Additionally, our results highlight the potential protective effect of ICSs in COVID‐19, suggesting that maintenance of regular COPD therapy during SARS‐CoV‐2 infection may be beneficial.

Our findings should be interpreted with several limitations in mind. Despite rigorous statistical adjustment through IPW and multiple sensitivity analyses, residual confounding cannot be entirely eliminated. Our data from two hospitals in Northern China may limit generalizability, particularly regarding local treatment protocols and patient demographics. Additionally, we were unable to stratify COPD patients by disease severity (GOLD stages) or phenotype (emphysema‐predominant vs. chronic bronchitis‐predominant), which, as demonstrated by Vogelmeier et al. [29], may influence COVID‐19 outcomes heterogeneously. Furthermore, we lacked data on long‐term outcomes beyond hospital discharge, and quantitative CT imaging data to assess the extent of lung involvement were not available, which limited our ability to fully account for radiological severity. These omissions may influence the interpretation of our adjusted analyses.

## 5 Conclusion

In contrast to findings from earlier COVID‐19 waves, our study shows that COPD is not independently associated with an increased risk of adverse outcomes in patients hospitalized with SARS‐CoV‐2 during the Omicron period. This finding represents a significant shift from the established risk profile of COPD in the COVID‐19 Delta period and highlights the dynamic nature of risk factors as the virus evolves. The altered association between COPD and outcomes during the Omicron wave likely reflects a combination of factors, including the variant's distinct tropism, treatment patterns, evolving clinical management approaches, and resource allocation.

Clinicians should consider these findings when assessing risk and making management decisions for COPD patients with Omicron infection, emphasizing the need for comprehensive clinical evaluation rather than relying solely on the presence of COPD as a risk stratification factor. Future research should focus on understanding the biological mechanisms behind this altered relationship, particularly regarding variant‐specific pathogenicity, immunological interactions, and treatment effects. Longitudinal studies examining outcomes across successive variant waves would further elucidate the dynamic nature of risk factors in this evolving pandemic.

## Author Contributions

Study design: R.T. and Y.Y. Data collection: S.W., F.Z., R.L., Y.Z., L.Z., W.T., and L.L. Statistical analysis: L.L. and R.T. Writing: L.L. and R.T. All authors reviewed the study design, data analysis and interpretation, and manuscript preparation. All authors have approved the final draft of the manuscript.

## Disclosure

The sponsors did not interfere in the study design; collection, analysis, or interpretation of data; writing of the report; and the decision to submit the article for publication.

## Ethics Statement

The Ethics Committees of the China‐Japan Friendship Hospital (no. 2023‐KY‐286) and Tangshan Gongren Hospital (no. 2024‐027) approved and waived the informed consent for this retrospective chart review study.

## Conflicts of Interest

The authors declare no conflicts of interest.

## Data Availability

The data that support the findings of this study are available from the corresponding author upon reasonable request.
